# Assessing the effects of the COVID-19 pandemic on new ART initiation and viral load access among children and adolescents living with HIV in West Africa: an interrupted time series analysis

**DOI:** 10.3389/fpubh.2025.1487302

**Published:** 2025-06-11

**Authors:** Yao Rodion Konu, Karen Malateste, Sophie Desmonde, Désiré Dahourou, Madeleine Amorissani-Folquet, Mariam Sylla, Caroline Yonaba, Lehila Tossa-Bagnan, Joycelyn Dame, Didier Koumavi Ekouevi, Valériane Leroy

**Affiliations:** ^1^Département de Santé Publique, Université de Lomé, Lomé, Togo; ^2^Université de Bordeaux, Institut National de la Recherche Médicale (Inserm) UMR 1219, Institut de Recherche pour le Développement (IRD) EMR 271, Bordeaux Population Health Centre, Bordeaux, France; ^3^Centre d’Epidémiologie et Recherche en Santé des POPulations (CERPOP), Inserm - Université Toulouse, Toulouse, France; ^4^Département de Santé Biomédicale et Publique, Institut de Recherche en Sciences de la Santé, Ouagadougou, Burkina Faso; ^5^Centre Hospitalier Universitaire (CHU) Cocody, Abidjan, Côte d’Ivoire; ^6^CHU Gabriel Touré, Bamako, Mali; ^7^CHU Yalgado Ouedraogo, Ouagadougou, Burkina Faso; ^8^Centre National Hospitalier Universitaire, Cotonou, Bénin; ^9^Departement of Child Health, University of Ghana and Korle Bu Teaching Hospital, Accra, Ghana

**Keywords:** HIV, COVID-19 pandemic, ART initiation, VL testing, West Africa

## Abstract

**Background:**

Before the COVID-19 pandemic, the pediatric continuum of HIV care lagged behind that of adults. The present study aimed to describe the effects of the COVID-19 pandemic on access to HIV care among children and adolescents (0–19 years) living with HIV (CALHIV) in five West African countries.

**Methods:**

Within this observational multicenter study, we conducted an interrupted time series analysis by including all antiretroviral therapy (ART)-naive CALHIV newly enrolled between 2018 and 2021. Two monthly documented outcomes were analyzed, namely, the number of ART initiators and the number of viral load (VL) tests performed. We fitted Poisson segmented regression models to estimate immediate changes at pandemic onset and per-pandemic trends through incidence rate ratios (IRRs) with 95% confidence intervals (CIs).

**Results:**

Immediately after the start of the pandemic, the average number of ART initiations decreased by 83.8% in Burkina Faso (IRR: 0.162; [95%CI: 0.043–0.609]) and 70.9% in Ghana (IRR: 0.291 [0.171–0.494]). Similarly, the number of VL tests performed decreased by 51% in Burkina Faso (IRR: 0.409 [0.253–0.662]). There were no significant trends in the number of ART initiations during the pandemic, except in Ghana (IRR: 1.146 [1.073–1.224]). The number of VL tests performed monthly in clinics in Côte d’Ivoire and Ghana decreased during the pandemic.

**Conclusion:**

ART initiation and VL testing activities were maintained in the majority of West African pediatric clinics, despite the COVID-19 pandemic and subsequent crisis. HIV care continuum monitoring in CALHIV should be maintained during the postpandemic period to identify and mitigate potential lasting effects.

## Introduction

Before the COVID-19 pandemic emerged in 2020, the pediatric continuum of HIV care lagged behind that of adults. Globally, in 2019, 68% of adults (≥15 years) living with HIV were receiving antiretroviral therapy (ART), whereas only 53% of children (≤14 years of age) were accessing treatment (1). To date, ART coverage in children is still well below the global UNAIDS target of 95% in all regions worldwide and even more in West and Central Africa (2).

Epidemics threaten global health efforts to fight HIV through direct (reduction in service delivery) and indirect (reduction in service use) mechanisms (3). For example, in 2014, the Ebola epidemic in West Africa led to a decline in access to and use of maternal and child health services (4, 5) and in HIV care for adults (6). This decline has led to increased morbidity and mortality in these populations (4, 6).

According to the Global Fund, the COVID-19 pandemic interrupted essential HIV testing, prevention and treatment care services, particularly among key and vulnerable populations in the countries supported by the Global Fund, especially sub-Saharan Africa (7). Between 2019 and 2020, voluntary medical male circumcision fell by 27%, and the number of people benefiting from an HIV prevention program fell by 11% (7). The number of HIV-positive mothers treated to prevent transmission of HIV to their babies decreased by 4.5%. Screening rates have decreased by 22%, leading to a decrease in antiretroviral treatment indicators in most countries (7, 8).

Health systems and HIV programs in Africa are characterized by shortages of trained medical personnel and financial constraints (9, 10). Given these and other weaknesses, initial projections of the effects of the COVID-19 pandemic were pessimistic, owing to the reallocation of resources needed to combat the COVID-19 pandemic, which could affect the response to other threats, such as malaria, HIV and tuberculosis (9).

Several studies have documented the extent of HIV treatment decline or interruption during the pandemic, most of which have focused on adult populations (3, 11–13). Some authors have even suggested that the indirect effects of the COVID-19 pandemic may have reversed decades of progress in improving health outcomes in Africa (14, 15). With respect to HIV outcomes, the effect of the pandemic on children and adolescents living with HIV (CALHIV) may be even worse than that on adults.

For CALHIV, the greatest survival outcomes can be achieved only with optimal, uninterrupted treatment with effective ART (16). Treatment disruptions, defined as any interruption or alteration of initial ART, may result from patient-level factors, provider-level factors, or systems-level factors (16). Unfortunately, treatment disruptions may result in treatment failure, the acquisition of resistance mutations, and the loss of future treatment options—which are particularly consequential in CALHIV (16). If inadequately treated, children and adolescents progress much faster to poor outcomes (low rates of viral suppression, high susceptibility to viral rebound, and poor rates of retention), AIDS and death (16). Therefore, when individuals are susceptible to interruption of care provisions, the care continuum of CALHIV should be closely monitored, and adequate adaptation strategies should be implemented.

Sub-Saharan Africa accounts for more than two-thirds (65%) of people living with HIV (17). In 2023, 5.1 million people were living with HIV in West Africa and Central Africa (18), while approximately 26% of CALHIV were registered in the same region (19). Among these CALHIV, only 37% knew their status, 37% were receiving ART, and 30% were virally suppressed (19). Little information is available on the effects of the COVID-19 pandemic on HIV care for children and adolescents, particularly in West Africa (20, 21). Such information would be useful in preparing for future shocks to the health system and in considering strategies to compensate for any delays before looking ahead to achieving the 95–95-95 targets (22). In the present study, we describe the effects of the COVID-19 pandemic on access to HIV care (ART initiation and HIV viral load [VL] testing) among new enrolled CALHIV aged 0–19 years in 8 clinical sites across five countries contributing to the West African International Epidemiology Databases to Evaluate AIDS (IeDEA) pediatric cohort.

## Methods

### Data source

The IeDEA Pediatric West African Database on AIDS (pWADA) collaboration is an international research consortium established in 2006 that gathers, harmonizes and aggregates routinely collected data on HIV and AIDS (23). Data from all CALHIV with confirmed HIV infection attending HIV care services in 10 West African pediatric clinics across 7 countries contribute to the pediatric pWADA database.^1^

Within this observational, multicenter study, we conducted an interrupted time series analysis (ITS) at clinical sites in Benin (*n* = 1), Burkina Faso (*n* = 1), Côte d’Ivoire (*n* = 4), Ghana (*n* = 1) and Mali (*n* = 1) by including data from all ART-naive CALHIV newly enrolled between January 2018 and June 2021. The HIV care continuum figures in CALHIV in these countries are consistent with those in the West African region. Additionally, the estimated number of CALHIV aged 0 to 19 years in the studied countries ranges from 9,000 in Benin to 31,000 in Cote d’Ivoire in 2023, according to UNICEF (24). The annual reported number of AIDS-related deaths varies from 500 in Benin to 1700 in Ghana (24).

We defined the prepandemic period until March 31, 2020, and the pandemic period from April 1, 2020, to the merger database closure date, which varied by site (Supplementary Table 1). Data from Togo and Nigeria were not included because they were incomplete.

### Outcomes and analysis

Segmented Poisson regression models were fitted to estimate the immediate effect of the pandemic compared with the prepandemic period and changes in trend (i.e., slope) during the pandemic period on the following two different outcomes documented monthly in each country: the number of new ART initiations and the number of viral load (VL) tests performed among newly enrolled CALHIV. These indicators are the sum of new ART initiations and the sum of HIV viral load tests performed in a month, respectively.

The regression model used the following equation:


$$l\mathrm{og}(E(Y))=\beta_{0}+\beta_{1}T+\beta_{2}L+\beta_{3}W$$

where Y represents the variable (indicator) of interest; T is the number of time points since the beginning of the observation period; L is an indicator variable equal to 1 for time points following the declaration of the COVID-19 pandemic (from April 2020); W represents the number of time points since the declaration of the pandemic (0 for the prepandemic period); *β0* represents the baseline level of the variable of interest; *β1* represents the average change in *log(E(Y))* per period before the COVID-19 pandemic; *β2* represents the average change in the level of *log(E(Y))* immediately after the occurrence of the COVID-19 pandemic (i.e., the change between the last measurement before and the first measurement the month after the interruption); and *β3* represents the average difference in slope between the COVID-19 pandemic period and the pre-COVID-19 pandemic period. The main parameters of interest, *β2* and *β3*, are presented and interpreted as incidence risk ratios (IRRs) to compare incidence rates before and during the pandemic with respective 95% confidence intervals (95% CIs).

Newey–West standard errors were used to account for heteroscedasticity and autocorrelation (25). We have previously used and fully described this statistical approach elsewhere (8). All the statistical analyses were performed using R version 4.2.2.

### Ethics statement

Each clinic obtained approval from their National Ethics Committees (Benin, N°26/MS/DC/SGM/CNERS/ST of February 2022; Burkina Faso, 2022–01-008; Côte d’Ivoire, 077-18/MSHP/CNESVS-km; Ghana, KBTH/MD/G3/24; and Mali, N°2018/78/CE/FMPOS). The analysis only used anonymized data collected from routine clinical care; thus, individual informed signed consent was waived.

## Results

A total of 612 CALHIV initiated antiretroviral treatment during the study period as follows: Côte d’Ivoire (*N* = 240, 39.2%), Mali (*N* = 153, 25.0%), Ghana (*N* = 111, 18.1%), Benin (*N* = 56, 9.2%), and Burkina Faso (*N* = 52, 8.5%). The age and sex distributions of the participants remained similar before and during the COVID-19 pandemic (Table 1).

**Table 1:** Description of baseline characteristics of pWADA children and adolescents initiating antiretroviral treatment before and during the COVID-19 pandemic period, January 2018 to December 2021.

Characteristic	Observation period (Months)		Total *N* = 612	*p*-value^1^
	Before the COVID-19 pandemic; *N* = 438	During the COVID-19 pandemic; *N* = 174		
Sex, N (%)				0.994
Male	239 (54.6)	95 (54.6)	334 (54.6)	
Female	199 (45.4)	79 (45.4)	278 (45.4)	
Age (y)				0.843
Median (25–75%)	5 (2–10)	5 (2–10)	5 (2–10)	
Range	0–19	0–18	0–19	
Country, N (%)				0.218
Benin	38 (8.7)	18 (10.3)	56 (9.2)	
Burkina Faso	41 (9.4)	11 (6.3)	52 (8.5)	
Côte d’Ivoire	181 (41.3)	59 (33.9)	240 (39.2)	
Ghana	74 (16.9)	37 (21.3)	111 (18.1)	
Mali	104 (23.7)	49 (28.2)	153 (25.0)	

1 Pearson’s Chi-squared test; Wilcoxon rank sum test.

### Immediate changes after the pandemic

Table 2 presents the effects of the COVID-19 pandemic on the number of new ART initiations and the number of VL tests performed.

**Table 2:** Effect of the COVID-19 pandemic on the number of new antiretroviral therapy initiation and viral load testing in five countries contributing to the IeDEA pediatric West African database to evaluate AIDS, Poisson segmented regression models.

	Level change before the COVID-19 pandemic declaration			Trend change during COVID-19 pandemic		
	IRR*	95% CI**	*p*-value	IRR	95% CI	*p*-value
Antiretroviral therapy initiation						
Benin	1.232	0.746–2.037	0.431	0.954	0.901–1.009	0.126
Burkina Faso	0.162	0.043–0.609	**0.019**	1.010	0.916–1.113	0.852
Côte d’Ivoire	0.825	0.629–1.082	0.190	1.062	1.032–1.093	**0.001**
Ghana	0.291	0.171–0.494	**0.001**	1.146	1.073–1.224	**0.002**
Mali	1.346	0.734–2.469	0.355	0.958	0.906–1.012	0.154
Viral load testing						
Benin	0.714	0.246–2.073	0.548	1.048	0.936–1.174	0.433
Burkina Faso	0.409	0.253–0.662	**0.003**	1.020	0.957–1.088	0.550
Côte d’ivoire	1.058	0.765–1.463	0.739	0.954	0.922–0.987	**0.018**
Ghana	2.108	0.951–4.671	0.091	0.776	0.693–0.870	**0.001**
Mali	2.655	1.207–5.836	**0.032**	0.949	0.897–1.005	0.097

*Incidence rate ratio; **95% confidence intervals. Bold *p*-values indicate statistical significance (*p* < 0.05).

Immediately after the start of the COVID-19 pandemic, the average number of ART initiations decreased significantly, with decreases of 83.8% in Burkina Faso (IRR: 0.162; [95% CI: 0.043–0.609]) and 70.9% in Ghana (IRR: 0.291 [0.171–0.494]). For the other countries, there were nonsignificant fluctuations before and during the COVID-19 pandemic (Table 1).

Similarly, the number of VL tests performed in April 2020 (immediately after the start of the pandemic) decreased by 51% in Burkina Faso (IRR: 0.409 [0.253–0.662]) compared with March 2020 (Table 1). Conversely, the number of VL tests doubled in Mali (IRR: 2.655 [1.207–5.836]).

### Pandemic trends

**Figure 1:**
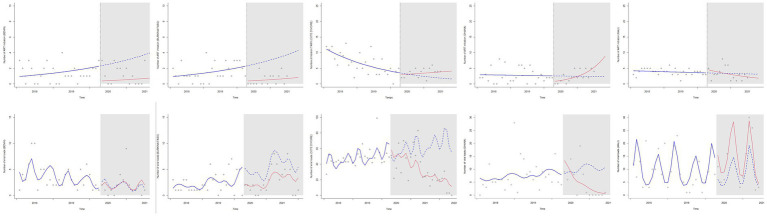
Trends in new antiretroviral treatment initiations and access to HIV viral load testing, before and after the COVID-19 pandemic declaration in the pediatric West African Database on AIDS, 2018–2021, Poisson segmented regression analysis. ART: antiretroviral treatment. The x axes are the calendar time axes. The y axes are the crude numbers of total services provided. The vertical black line shows the beginning of the COVID-19 pandemic. The blue line is the temporal trend in the pre pandemic period. The blue dots are the counterfactual scenarii estimated if the pandemic did not occur. The red line is the observed temporal trend after the pandemic was declared.

Figure 1 shows the observed and predicted trends over the study period. There were no significant trends in the number of ART initiations, except in Ghana. During the pandemic and after an initial drop, the number of ART initiations gradually increased monthly compared with that in the prepandemic period (IRR: 1.146; 95% CI: 1.073–1.224; Table 1).

Regarding the number of VL tests performed, pWADA clinics in Côte d’Ivoire and Ghana experienced a monthly decline during the pandemic period (Table 1).

## Discussion

The present study analyzed the effects of the COVID-19 pandemic on two routinely monitored outcomes among newly enrolled CALHIV in eight HIV clinics across five West African countries. The majority of pediatric HIV care clinics included in our analysis have been resilient with respect to ART initiation and VL testing.

### ART initiation

Only clinics in two countries (Burkina Faso and Ghana) out of five experienced a significant decrease in the number of new ART initiations at the start of the pandemic, followed by a status quo or positive trend in the following months. According to a separate site assessment survey across the global IeDEA consortium, only 6% of sites offering ART initiation services reported suspending these services after the onset of the pandemic (26). Other studies in West Africa have shown that mobility restrictions during the pandemic and the fear of being infected with COVID-19 limited the access of mothers and therefore children to health facilities, which may have delayed the initiation of ART and other care, such as screening (8).

As part of the solution, 30% of the global IeDEA sites reported that extending same-day ART initiation was a coping measure, which may explain why these sites had a status quo or positive ART initiation trend in subsequent months (26). Therefore, same-day ART initiation may be a relevant approach to achieve HIV treatment targets in children and adolescents or to maintain new ART initiation during a crisis that disrupts health care.

The specific organization of each clinic that provided data for this study during the pandemic may explain the observed differences. In a similar study within the adult cohort of the IeDEA consortium, Ben Farhat et al. reported that some sites in Côte d’Ivoire (including two participating in our study) set up community-based ART distribution mechanisms to adapt to the pandemic (27). This strategy, which has helped maintain ART initiation and dispensation, should be considered, at least in crisis situations. According to the global IeDEA consortium, 56% of sites experienced increased use of telemedicine (i.e., consultations by phone/web) in HIV-related care during the COVID-19 pandemic (26), and only 35% of the low-income countries experienced this increase in the use of telemedicine (26). Thus, the use of telemedicine had a negligible role in the present results. Although telemedicine remains relevant and is worth considering for maintaining care in times of crisis, its implementation is subject to logistical challenges in this region.

### VL testing

Measuring the viral load is essential for monitoring adherence to ART and assessing viral suppression, which is one of the therapeutic objectives. All PEPFAR-supported countries have reported a reduction in VL testing coverage, with the largest decrease during the March–May 2020 period, probably due to limited access to clinical and laboratory services at the early stage of the pandemic (28). Similarly, our analysis revealed that the number of VL tests conducted significantly decreased at the onset of the pandemic in Burkina Faso. Furthermore, we observed a downward trend in VL testing in Côte d’Ivoire and Ghana during the pandemic period, which may be explained by interruptions in HIV VL testing, including suspension of testing (22%), longer turnaround times (41%) and supply/reagent stockouts (22%), as reported at the global IeDEA consortium sites (26). Importantly, during the COVID-19 pandemic, manufacturers of VL testing platforms developed molecular diagnostic capabilities for SARS-CoV-2 using the same equipment used for HIV VL testing, and many laboratory staff members were shifted from molecular testing for HIV to testing for SARS-CoV-2, causing the decrease in HIV VL testing coverage (22). To reduce these adverse effects of responding to health emergencies, the response should also promote the continuity of ordinary health care services.

The impacts of the COVID-19 pandemic on health care services may vary from one country to another, with variations depending on the robustness and capacity of a country’s health care system before the pandemic and the intensity of response measures (27, 29). However, this type of study remains relevant, as it helps to prepare CALHIV management programs for future crises that could interrupt the provision of health services.

### Strengths and limitations

While other studies have assessed the effects of the Ebola (6) and COVID-19 (27) epidemics on adult cohorts in the IeDEA collaboration, the present study focused on children and adolescents using time series analysis methods. The impact of COVID-19 may have been minimized, as the pediatric clinics assessed were urban and referral sites, and in most countries, only one clinic contributed. Nevertheless, the present method allowed trends in the West African context to be explored. Although the present results cannot be extrapolated to all of sub-Saharan Africa, they remain consistent with two other analyses conducted in Nigeria (21) and Mozambique (20). This work, coupled with qualitative interviews with site workers, provides useful insight into the context and best practices for CALHIV management.

## Conclusion

The majority of West African pediatric HIV clinics included in this analysis have coped well with the COVID-19 pandemic and subsequent crises, particularly with respect to new ART initiation and the number of VL tests performed. Given that health crises are likely to occur in the future, it is essential to conduct analyses that incorporate qualitative methods to better understand the context and perspectives within pediatric HIV care services to improve preparedness and resilience. In the meantime, community-based ART delivery should be considered to ensure ART initiation and adherence, whereas reflections should address strengthening lab capacities for VL monitoring even during health crises. HIV care continuum monitoring in CALHIV should also be maintained during the postpandemic period to identify and mitigate potential lasting effects.

## Data Availability

The data analyzed in this study is subject to the following licenses/restrictions: the datasets used and/or analyzed during the current study are available from the corresponding author upon reasonable request. Requests to access these datasets should be directed to rodionko@yahoo.fr.

## Footnotes

^1^ http://iedea-wa.org/
