# Supplementary material for: Assessing the effects of the COVID-19 pandemic on new ART initiation and viral load access among children and adolescents living with HIV in West Africa: an interrupted time series analysis
Source: Front Public Health. 2025 Jun 11;13:1487302. doi: 10.3389/fpubh.2025.1487302 (PMC12187821; doi:10.3389/fpubh.2025.1487302)
Supplement: Supplementary file 1 [file Table_1.DOCX]

**Supplementary table 1:** Description of pWADA clinics included

| **CLINIC** | **NAME** | **OPEN DATE** | **CLOSURE DATE** | **COUNTRY** | **CITY** |
| --- | --- | --- | --- | --- | --- |
| CEPREF | Centre de Prise en Charge, de Recherche et de Formation | 27/04/1998 | 14/06/2021 | Côte d’Ivoire | Abidjan |
| CHUC | Centre Hospitalier Universitaire de Cocody | 26/02/1996 | 15/10/2021 | Côte d’Ivoire | Abidjan |
| CHUY | Centre Hospitalier Universitaire de Yopougon | 24/03/2000 | 12/05/2022 | Côte d’Ivoire | Abidjan |
| CHUYO | Centre Hospitalier Universitaire Yalgado Ouédraogo | 30/11/1999 | 15/04/2022 | Burkina Faso | Ouagadougou |
| CIRBA | Centre Intégré de Recherche Bioclinique d'Abidjan | 29/03/1995 | 29/03/2022 | Côte d’Ivoire | Abidjan |
| CNHU | Centre National Hospitalier Universitaire, Cotonou | 19/09/1998 | 08/07/2021 | Bénin | Cotonou |
| GABRIEL | Hôpital Gabriel Touré | 29/02/2000 | 10/07/2021 | Mali | Bamako |
| KBTH | Korle-Bu Teaching Hospital | 11/03/2000 | 13/07/2021 | Ghana | Accra |
